# Laser-driven low energy electron beams for single-shot ultra-fast probing of meso-scale materials and warm dense matter

**DOI:** 10.1038/s41598-023-30995-0

**Published:** 2023-03-14

**Authors:** Katerina Falk, Michal Šmíd, Karel Boháček, Uddhab Chaulagain, Yanjun Gu, Xiayun Pan, Pablo Perez-Martin, Miroslav Krůs, Michaela Kozlová

**Affiliations:** 1 Helmholtz-Zentrum Dresden-Rossendorf, Bautzner Landstraße 400, 01328 Dresden, Germany; 2 Technische Universität Dresden, 01062 Dresden, Germany; 3 Institute of Physics CAS, 182 21 Prague, Czech Republic; 4 ELI Beamlines Facility, The Extreme Light Infrastructure ERIC, 25241 Dolní Břežany, Czech Republic; 5 Institute of Scientific and Industrial Research, Osaka University, Ibaraki, Osaka 567-0047 Japan; 6 Institute of Plasma Physics CAS, 182 21 Prague, Czech Republic

**Keywords:** Imaging techniques, Laser-produced plasmas, Plasma-based accelerators

## Abstract

Laser wakefield acceleration has proven to be an excellent source of electrons and X-rays suitable for ultra-fast probing of matter. These novel beams have demonstrated unprecedented spatial and temporal resolution allowing for new discoveries in material science and plasma physics. In particular, the study of dynamic processes such as non-thermal melt and lattice changes on femtosecond time-scales have paved a way to completely new scientific horizons. Here, we demonstrate the first single-shot electron radiography measurement using an femtosecond electron source based on the downramp-density gradient laser-wakefield-acceleration with the use of a compact Ti:sapphire laser. A quasi-monoenergetic electron beam with mean energy of 1.9 ± 0.4 MeV and charge 77 ± 47 pC per shot was generated by the laser incident onto a gas target and collimated using a two ring-magnet beam path. High quality electron radiography of solid objects with spatial resolution better than 150 $\upmu$m was demonstrated. Further developments of this scheme have the potential to obtain single-shot ultrafast electron diffraction from dynamic lattices. This scheme poses a great promise for smaller scale university laboratories and facilities for efficient single-shot probing of warm dense matter, medical imaging and the study of dynamic processes in matter with broad application to inertial confinement fusion and meso-scale materials (mg g/cm$^2$).

## Introduction

With the rise of high energy laser and accelerator facilities with ultra-short pulse capabilities new horizons for the study of dynamic behaviour of exotic materials and dense plasmas have opened. In harmony with decades of development of high precision electron radiography, diffraction and microscopy, these probes now promise robust and powerful diagnostic techniques to determine material structure in equilibrium as well during dynamic changes with femtosecond to picosecond time resolution all the way down to atomic scale. Specifically, the emergence of novel particle acceleration schemes based on intense ultra-fast (femtosecond duration) lasers have paved a new way to obtain real-time measurement of exotic processes such as bond-hardening or nonthermal melt of lattices^1–4^. One of the most promising approaches is to use electron and x-ray beams from laser wakefield acceleration (LWFA) driven by lasers with duration of 30–100 fs^5,6^. While such short electron pulses can be achieved also with accelerators or by more compact radio-frequency (RF) guns and cavities^7,8^, the accelerator sources are very large with very limited user access and the RF technology has intrinsic limitation in the pulse duration and high timing jitter, which makes reaching resolution below 100 fs challenging. The LWFA electron sources readily achieve few 10s of fs temporal resolution thanks to the short duration of the drive laser. Several proof of principle electron radiography (eRad) measurements have been carried out with LWFA sources^9–13^. LWFA eRad diagnostic has shown the ability to provide high resolution imaging of biological samples^10,12^ as well as ultra-fast imaging of rapidly evolving magnetic fields with fs temporal resolution^11^. LWFA electron sources have also demonstrated ultra-fast measurement of dynamic changes in proto-excited silicon lattice using electron diffraction^14^. The advantage of LWFA sources is not only their relative compactness, but also the potential to combine it with drive lasers that can produce large amounts of warm dense matter or shock-waves in solid materials inducing dynamic structural changes. The laser systems capable of LWFA are often based at laboratories with multi-beam facilities or themselves can be split into pump-and-probe style setups, allowing precise jitter-free synchronization between the drive and probe beams^11,14^.

To date most electron probes have operated at high energies due to the nature of the acceleration mechanisms used. Electrons above $\sim 30$ MeV however generate large amounts of bremsstrahlung radiation during the interaction with large nuclear electric field resulting in substantial energy loss in the probe in just a few interactions posing limits on the diagnostic^15^. The interaction between the high energy electron probe beam and studied sample is dominated by multiple Coulomb scattering further complicating the data analysis. In the MeV energy range however, the energy loss of the electron in a medium is mainly due to ionisation processes making it sensitive to the areal density distribution of the object and less sensitive to the energy spread of the beam. Moreover, the MeV energy range is perfect for electron diffraction as it can provide the high resolution patterns on the detector placed only a few m away from the source for the atomic scales, making it very compact and well suited for practical use in a typical laboratory.

Laser produced electron beams with the energy in the order 0.5–2 MeV are thus very useful for diagnostic purposes during complex laser-matter interaction experiments. They might be used for example for electron diffraction or scattering, where the significantly higher cross-section for electrons gives these beams an advantage compared to X-rays, or for electron radiography (eRad). Electrons with low energy and small rest mass result in a system with very high sensitivity to thin probed samples (mm level) as the probing length is limited by the range of the charged particle in the material. The MeV energy range for the electron beams is advantageous for the probing of warm dense matter (WDM), inertial confinement fusion (ICF) implosions, imaging of meso-scale materials (mg g/cm$^2$), magnetic fields or biological samples as they keep the perturbation of the probed sample by the beam to the minimum^10,15–18^. The production of intense MeV electron beams can be achieved via either so called downramp-density gradient laser-wakefield-acceleration (DDG-LWFA)^6,19^, or via ponderomotive acceleration^20–22^. Reliable measurements have been carried out with kHz DDG-LWFA sources driven by 10 mJ lasers, however due to relatively small electron charge achieved in a single shot, accumulation of multiple shots is needed in this case^14,16^. This is favourable in applications where the high-repetition rate can be utilised, but when single-shot measurement in dynamic systems is required a system with higher electron charge is required. In order to achieve single-shot eRad measurement, high charge electron beams are needed. The highest electron beam yields suitable for MeV-class eRad have been achieved with kJ-class lasers using the self-modulated LWFA mechanism^17,23^. However, most smaller laboratories have access to only few 100’s mJ class fs lasers. MeV electron beams reaching up to 100 s of pC in charge have also been generated by improper trapping in LWFA, however they result in high divergence ring structures around a collimated central high energy electron beam which are not likely candidates for probing^24,25^.

In this work we present the first single-shot eRad measurement based on the DDG-LWFA scheme. Our compact system is capable of generating bright MeV electron beams with relatively low laser energies that is accessible to even small scale laboratories and universities. A too low energy electron beam suffers from a strong temporal dispersion. However, MeV-class electron beams are easy to manipulate with small size magnets allowing for selection of a narrow energy band of electrons, which in turn improves the eRad resolution by reducing the energy spread associated with the energy loss in the sample and chromatic aberrations in the magnetic lens imaging system^26^. This source is suitable for single-shot MeV electron radiography measurement with a monochromatic electron output utilising a simple and robust magnetic lense system. This method also offers unprecedented temporal (fs) and spatial (100 s of $\upmu$m) resolution and can be sychronized with additional laser drivers making it an ideal candidate for studying dynamic processes in materials and electromagnetic fields.

**Figure 1:**
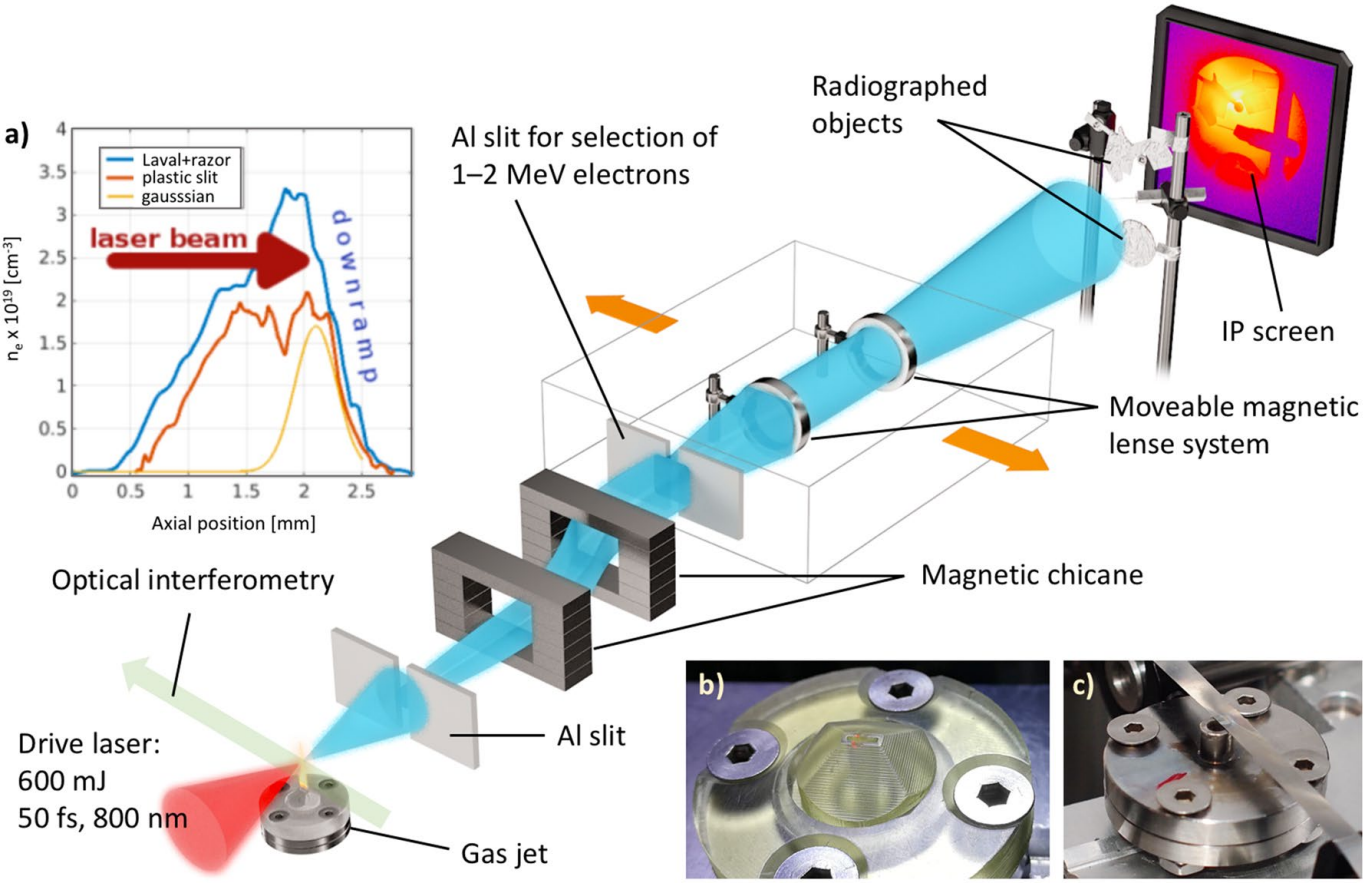
Experimental setup at the Ti:Sa laser system at the PALS facility. The setup was fit in a vacuum chamber with an attached smaller chamber for extending the electron beam path with an imaging plate placed 267 cm away from the gas nozzle. A set of Al and steel objects of various shapes and thicknesses was placed $\sim 5$ cm before the imaging plates for a radiography measurement. A narrow band of electron energies was selected by an Al slit coupled with a dipole magnet and collimated by a set of ring magnetic lenses that conveyed the monochromized electron beam onto the IP. The ultra-fast interferometric measurement was carried out by the 1st harmonic split off component of the main laser pulse directly from the compressor with a known delay to the main pulse. Inserted figures show a comparison between density profiles of the plastic slit and Laval nozzles (**a**), and the photographs of the gas jet nozzles: (**b**) 3D printed plastic 500 $\upmu$m slit nozzle; (**c**) laval metallic nozzle with a razor blade to produce a steep density gradient on the rear side of the gas profile.

## Experimental setup

Experiments were performed during several experimental campaigns at the Ti:Sapphire laser system at the Prague Asterix Laser System (PALS) facility in Prague, Czech Republic. The schematic of the experiment is shown in Fig. 1. The laser pulse reached energy 600 mJ with ultra-short pulse duration 50 fs and a gaussian temporal profile at 800 nm wavelength. The beam was then focused by a 30$^{\circ }$ off-axis parabola (OAP) with f/8 to the best focus of $\sim 10$ $\upmu$m diameter, providing incident laser intensity of $1.5 \times 10^{19}$ W/cm$^{2}$ with the normalised vector potential a$_0 = eE_0/m_ec\omega _0= 2.7$. The target was a gas jet with He:Ar mixture (99:1 volume ratio). An array of different nozzles with various sizes, materials were used to shape the gas column density gradient. These included 3D printed plastic slit aperture nozzles of various sizes, 100 $\upmu$m diameter glass capillary and a standard Laval type supersonic circular nozzle with 3 mm diameter. Supersonic metallic narrow slit nozzles were also tested at higher backing pressures above 100 bar. In some configurations a metal razor blade was placed on top a Laval metallic nozzle at the back of the gas column in order to produce a sharp downward density gradient in the gas. The resultant plasma peak densities ranged from 4 $\times 10^{18}$ to $5 \times 10^{19}$ cm$^{-3}$ depending on the nozzle geometry and backing pressure. The plasma density profile was measured in a transverse direction using a Mach-Zehnder interferometer. The electron beam was first characterised by a dipole magnet serving as an electron spectrometer coupled with a set of Lanex screens and CCD cameras. The spatial profile of the beam was restricted by 5 mm wide Al slit at the entrance to the magnetic spectrometer in order to reduce the electron beam divergence and improve the energy resolution. This magnet was then replaced with a magnetic chicane consisting of two separate blocks spatially offseting trajectories of electrons with different energies as shown in Fig. 1. Another Al slit was placed behind the chicane on a translational stage providing an option to select a narrow energy band of the electron beam. Since the typical large divergence of these low energy electron beams from LWFA source poses a drawback for many applications, the setup also included the possibility of focusing and collimation of the electron beam using a pair of ring magnetic lenses as shown in Fig. 1. This pair of lenses was coupled with the 5 mm wide exit slit and their focusing power and alignment were matched to the selected energy band corresponding to 1.9 ± 0.4 MeV. The electron beam spatial profile and pointing was measured by another large Lanex screen with an optical CCD camera further down-stream. Radiographic image of various metal objects and electron charge was collected by a Fujifim MS imaging plate (IP) placed 267 cm away from the gas nozzle, which was the electron beam source.

## Results

### Optimisation of the electron beam

**Figure 2:**
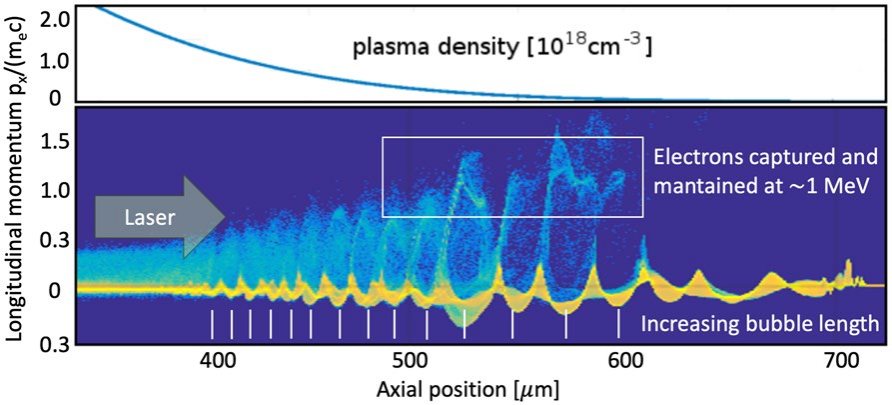
Electron phase-space diagram from the PIC simulation^27,28^. The y-axis corresponds to the longitudinal momentum $p_x/m_{e}c$. The negative values thus correspond to the particles move against laser propagation direction.

The objective was to achieve a large number of low energy (0.5–2 MeV) electrons in the probe beam. Such low energy electrons are easy to manipulate with cheap and small magnets, making it simple to select narrow energy bands as well as manipulating the beam path producing a parallel electron beam useful for imaging purposes. Moreover, by tailoring the LWFA mechanism to favour these low energies, we are increasing the efficiency of the energy conversion mechanism with the limited laser power available. The system was optimised to favor the DDG-LWFA scheme^6,14^. For this injection mechanism the laser needs to be focused into a steep downward density gradient on the backside of the gas column. In order to support this experiment, 2D particle-in-cell (PIC) simulations were carried out using the Epoch code^27,28^, see Fig. 2. In the simulation, the laser beam was let to propagate through a decreasing density profile resulting in an increased plasma wavelength and with that the wakes were getting longer and the phase velocity increases. In this scheme, many electrons enter the wake on the rear side, but at the decreasing density gradient the electrons are accelerated to much lower energies due to short dephasing length. These large wakes are driven by the same laser energy, they can thus accelerate a greater number of electrons producing a high charge low energy beam. Different sets of PIC simulations with varied downramp-density gradient were carried out to test the sensitivity of the acceleration scheme. They have shown that the peak energy of the accelerated electrons remains unchanged with varied density profile gradient, while injected charge is lower for steeper gradients. The DDG-LWFA scheme thus appears to be rather robust with respect to the peak energy of the resultant electron beam and shallower gradients can result in higher intensity.

**Figure 3:**
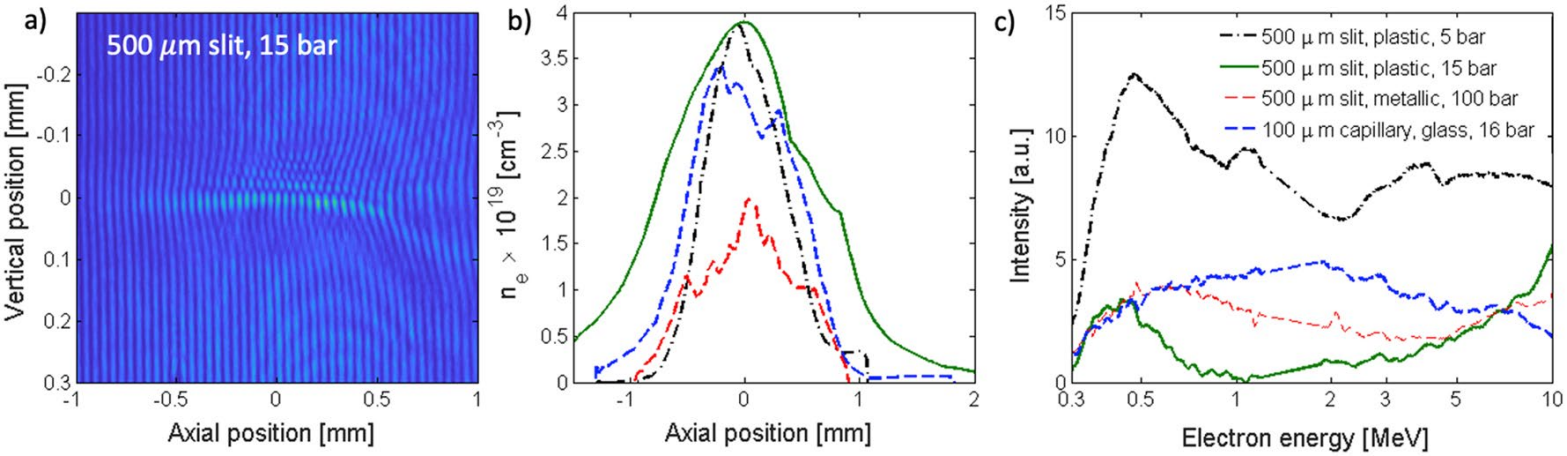
Optimisation of the electron beam with changing nozzle topology, laser focus position and backing gas pressure: (**a**) an example of interferometry image for 500 $\upmu$m plastic slit nozzle at 15 bar, (**b**) electron density measurements, and (**c**) electron spectra respectively.

In order to optimize the electron beam, interferometry measurement of the plasma density profile along the laser axis was compared with the resultant electron spectra obtained by the magnetic spectrometer. Combining these two measurements provides a clear relationship between the electron density and accelerated charge. The backing gas pressure, nozzle topology and laser focus position were varied testing different acceleration parameters. The electron density and electron spectra measurements for the 500 $\upmu$m slit 100 $\upmu$m capillary nozzles are shown in Fig. 3. In each configuration the position of the nozzle was varied with respect to the laser focus to find the best position for electron acceleration in the desired regime. For all gas backing pressures, $4 \times 10^{18}$ cm$^{-3}$ was the cut-off electron density in the plasma channel, below which barely any accelerated electrons were seen. Crossing this threshold the accelerated charge rose sharply. The 3 mm diameter Laval nozzle coupled with metal razor blade provided comparable results with the 3D printed 500 $\upmu$m slit nozzle. While the plasma profile was longer ($\sim 1.5$ mm), it was more stable and durable than the 3D printed plastic slit nozzle. For higher pressures (15 bar), the nozzle had to be put further from the laser and the longitudinal focus position had to be shifted upstream to compensate for the gas expansion, producing longer channel more suitable for classical LWFA. When the backing gas pressure was decreased, the laser had to be focused closer to the nozzle opening and the ponderomotively accelerated electrons started to dominate the spectra and the LWFA peak was below 10 MeV. By these means, the intensity of electrons below 5 MeV increased. The use of high backing pressure (100 bar) with 500 $\upmu$m metallic slit nozzle produced inhomogeneous density profile and a broad electron spectrum. The 100 $\upmu$m capillary nozzle produced a very sharp density profile, but the electron spectrum was broad and very low intensity. Here, shots with lower backing pressure produced higher electron charges. At optimised position of the laser focus with respect to the capillary nozzle, the electron densities of $3\times 10^{19}$ cm$^{-3}$ were generated in the plasma channel leading to the conclusion that the higher electron charge is due to shorter gas length. For longer gas profiles, the electron charge can be destroyed either due to laser-wakefield dephasing or by decay of the accelerating bubble. However, the overall intensity of this beam was rather low. The optimal conditions have been found with the 500 $\upmu$m slit nozzles and the 3 mm diameter Laval nozzle coupled with metal razor blade, which both provided a sufficient density gradient for the DDG-LWFA to take place, while also keeping a contribution from ponderomotively accelerated electrons and sufficiently long plasma profile for a large number of electrons to be accelerated. The ponderomotive acceleration was much less sensitive to the position of the laser focus.

The more durable 3 mm Laval nozzle coupled with metal razor blade was selected for the further operation and eRad measurements. The electron beam produced reached energies up to $\sim 20$ MeV with peak around 0.5–2 MeV in the DDG-LWFA regime^29^. Prior the magnet the beam divergence was found to be 20 mrad^30^ and the total electron charge was estimated to be up to 5 nC in the whole electron beam. The typical electron spectra measured by the dipole magnet are shown in Fig. 3b. This beam was then let into the magnetic chicane as shown in Fig. 1, which employed the 5 mm wide Al slit and a set of magnetic lenses to select a narrower energy range around 1.9 MeV electron energy and collimate the electron beam. The electron charge was only measured for the selected energy range as discussed below.

### Electron radiographic measurement

**Figure 4:**
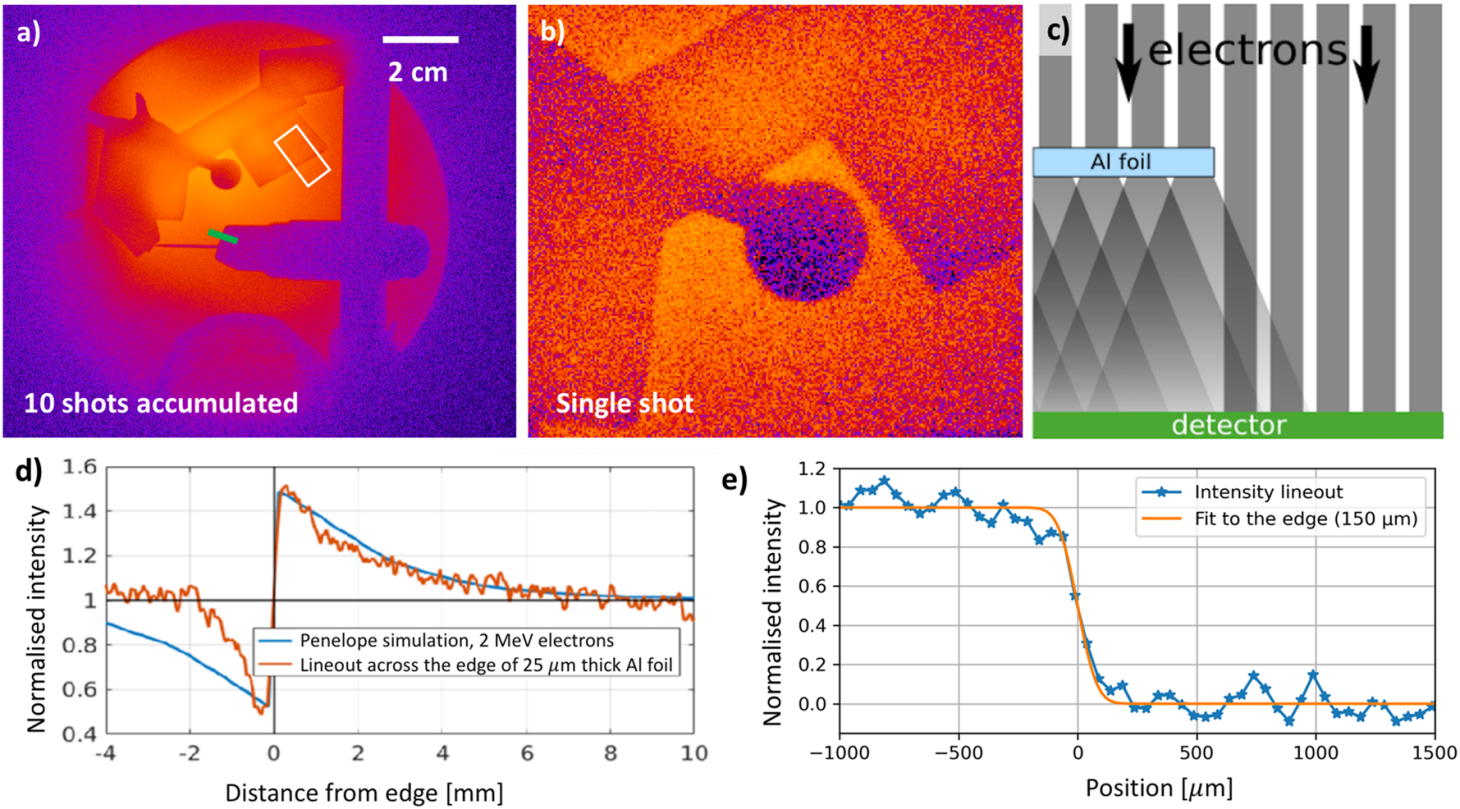
Radiography measurement: (**a**) full IP field of view with 10 shots accumulation, (**b**) enlarged detail of a single shot image, (**c**) schematic image showing the contrast enhancement on the Al foil edge, (**d**) comparison of the contrast enhancement of the edge compared with the Penelope simulations, and (**e**) intensity lineout across a sharp edge used to estimate the spatial resolution. The area marked by the white rectangle in image (**a**) signifies the region of interest, which was used for the contrast enhancement analysis and the thick green line shows where the intensity lineout used for the spatial resolution estimate was taken.

The electron beamline was optimized by changing the lateral offset of the ring magnets, which selects the energy range of the electrons for the radiographic measurement. This was first performed online with a Lanex screen and once the optimal distance was found, the Lanex was replaced with an IP. At this point, the incident electron beam was quasi-parallel after passing through the magnetic lens assembly and thus the radiographed object can be placed far away from the source reducing background in the eRad measurement. At the same time, the collimation of the electron beam means that there is no magnification in the measured image. The stopping range of 1–2 MeV electrons is $\sim 2$ mm for Al, $\sim 1$ mm for steel, and 4–5 mm in water respectively. Various steel and aluminium objects with the thickness ranging from 12 $\upmu$m to $\sim$ cm were placed in the beam $\sim 5$ cm in front of the IP, which recorded the eRad images. An image with a range of radiographed objects with 10 shots accumulation is shown in Fig. 4a. Sharp images were obtained even in the single-shot operation, see Fig. 4b. The width of the edge of sharpest object seen on single-shot radiograph is $\sim 150$ $\upmu$m, i.e. 3 pixels on the IP detector as shown in Fig. 4e. This number serves as an estimate of achievable spatial resolution.

Using absolute calibration of the Fujifim MS imaging plate has yielded 770 pC of total electron charge integrated over 10 shots, i.e. an average charge of 77 pC per laser shot^31,32^. It should be noted that this is the charge of electrons only in the narrow range around 1.9 MeV energy, thus only a fraction of the total electron charge in each shot. The shot to shot fluctuations of the charge were measured to be 61%. Therefore the uncertainty of the charge measurement can be assessed as $77 \pm 47$ pC. This result is in a good agreement with the PIC simulations that predicted total charge in the beam integrated over all electron energies of 7 nC, which corresponds to estimated $\sim 175$ pC after propagation through the beam path. Our eRad measurements show that with the energy discrimination with the magnetic lens assembly, this is sufficient for single-shot measurement producing sharp images with good spatial resolution, which is also suitable for the study of dynamic processes. A strong edge enhancement effect can be seen in the radiographic images, see Fig 4c,d. This enhancement in the contrast across the foil edge, marked by the red curve in 4d, is caused by weak scattering of electrons penetrating through the thin foil. In order to analyze the eRad images, Penelope Monte-Carlo simulations were carried out^33^. A special focus was put on reconstructing the edge effect. A set of simulations with varied input electron beam energies penetrating 25 $\upmu$m thick Al foil were carried until the best agreement was found for 2 MeV electrons. The plot comparing the Penelope simulation result with the intensity lineout across the 25 $\upmu$m thick Al foil from a single shot eRad measurement can be seen in Fig. 4d. This result shows an excellent agreement with the spectroscopic measurement and independently confirms the mean electron energy in the electron probe beam.

## Discussion and conclusions

**Figure 5:**
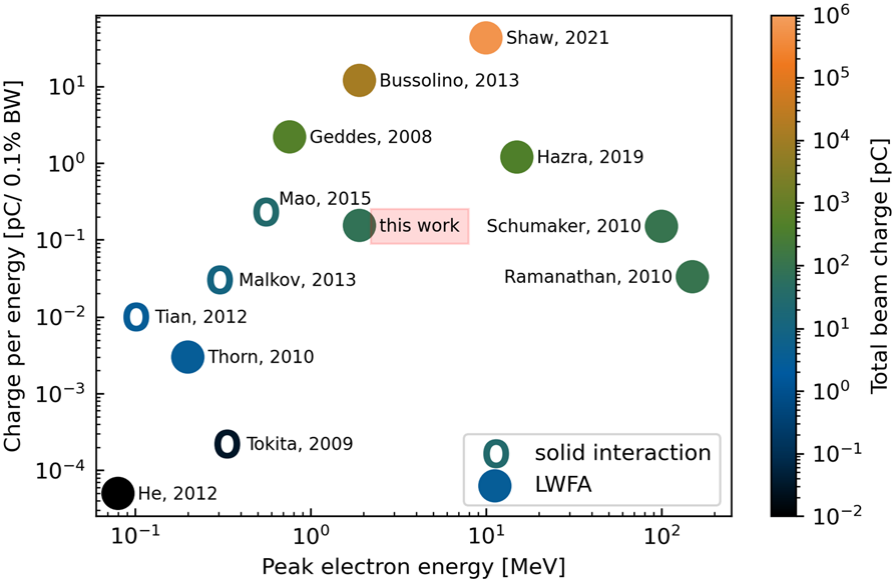
Charge per unit energy for various laser-driven electron sources. The LWFA electron sources^6,10–13,17,19,34^ are marked by solid color circular markers and other electron sources based on different schemes of laser interaction with solids^35–38^ are shown as empty circles. The colorbar scale then compares the drive laser intensities.

In conclusion, the first single shot electron radiography of static objects has been demonstrated using an electron beam with mean energy of 1.9 ± 0.4 MeV and charge 77 pC/shot generated by a 50 fs short-pulse laser through downramp-density gradient LWFA mechanism. In order to produce a clear eRad image, a narrow energy range of the electrons was selected by a magnetic chicane coupled with a slit and the resultant beam was collimated by using a two ring magnet system producing a well optimised parallel probe beam suitable for ultra-fast applications. The measured scattering edge enhancement confirmed the narrow energy spread of the beam. The spatial resolution of these images was found to be better than 150 $\upmu$m and could be further improved by optimizing the beamline. The applicability of this electron source was assessed by comparing its charge per unit energy to other similar laser-driven electron beams, which is shown in Fig. 5. Only few works based on LWFA have reported comparably high charge at desired beam energies and all of those reporting higher charge make use of much bigger laser systems capable of delivering more energy on target^6,10–13,17,19,34^. Our results are also compared with other laser-based acceleration schemes with solid targets^35–38^. LWFA sources are more favourable for probing of matter due to easier target replacement and lower debris production. The beam divergence and energy tunability can be improved by a better beam-path manipulation and the potential use of electromagnetic solenoids^16^. Furthermore, a more stable electron source with a better optimised magnetic focusing can be achieved with more laser energy and better pointing stability. With further sample optimisation this probing method can be utilised for single-shot electron diffraction or for the study of transient phenomena such as non-thermal melt of materials, bond hardening or softening, material deformation in shockwaves, phase transitions or transport properties and structure of WDM. In comparison with compact kHz sources^14,16,19^, this scheme allows for single-shot measurements thanks to the relatively high final electron charge, which is particularly important in dynamic experiments where accumulation of multiple shots is not possible for various reasons including large shot-to-shot variation or a low number of available shots such as ICF research.

## Methods

### The gas targets

The plastic slit nozzles were 3D printed using the Stereolithography (SLA) Anycubic Photon printer with 47 $\upmu$m accuracy using Translucent UV Resin monomer and photo initiator. This nozzle design had a smooth connection between the valve and the exit window to allow non-turbulent gas flow, therefore enhancing the gas profile. The 100 $\upmu$m diameter capillary was manufactured from fused silica using a hybrid 3D laser machining technique^39^. Fast removal nanosecond rear-side processing was implemented for the frame manufacturing, and Femtosecond Laser-assisted Selective Chemical Etching (FLSE) technique was used for the high-precision micrometric channel formation. The gas target was positioned on a target tower with x-y-z translational motions to adjust the position of the nozzle with respect to the laser focus. In the presented configuration, different backing gas pressures (5, 7, 10, 15 and 20 bar) were used to control the resultant plasma density profile. The optimal backing pressures for the 500 $\upmu$m slit nozzle was found to be 5 bar and for the 3 mm diameter Laval nozzle with razor it was 20 bar respectively. For each pressure, the position of the nozzle was varied in order to find its optimum in sense of maximal electron acceleration. The gas release system included a fast valve switch limiting the gas release into the chamber protecting the compressor and other sensitive equipment. Differential pumping system was employed to speed up the pumping cycle so that the build up of pressure in the vacuum chamber was minimized. The electron density in the laser channel was extracted from the fringe shift in the interferograms obtained by the Mach-Zehnder system, using Abel inversion along the horizontal axis assuming cylindrical geometry.

### The magnetic spectrometer and electron beam manipulation

The electron spectra for the various gas target configuration were first examined by a classical electron spectrometer. This consisted of dipole magnet with strength 78 mT and size $7 \times 7$ cm and the electrons inside and behind the magnet were detected by a Lanex screen. After the best experimental configuration was found, this magnetic spectrometer was replaced with a magnetic chicane, Al slit and two magnetic ring magnets, which were then used to select the desired part of the electron spectrum and produce a collimated beam for electron radiography. The chicane consisted of two blocks, each was 2 cm wide and 7 cm long, with the spacing of 2 cm as shown in Fig. 1. The maximum field inside each magnet was 75 mT. It parallelized electrons in the range of 0.3–3 MeV without destroying the beam. This dipole magnetic spectrometer system offset the electron trajectories by an amount depending on their energies, but it kept their direction parallel to the experimental axis. This feature enabled adjustment of energy selection and utilization of the beam downstream. Behind the magnets, there was a removable 5 mm wide slit and a removable Lanex screen. Two modes of operation were possible. With the Lanex screen inserted, the reference electron spectrum was measured. When the screen was out, it was possible to select electrons with a desired energy range using the Al slit. In this mode the online reference measurement of the electron spectrum was still available as the edge of the Lanex screen was always kept partially inserted in the beam. The resultant quazi-monoenergetic beam was then collimated by a pair of Neodymium magnetic rings with 22 mm diameter. An axial field of each magnet was measured. An electron ray-tracing simulation was used to determine the focal length of each ring magnet and to design the electron beamline. This beam forming system acted as a set of magnetic lenses, which focal length was a function of the electron energy. Different electron energies could be selected by lateral translation of the slit and the ring magnets. A single magnetic lens system for electron focusing only a narrow range of electron energies was also tested. A Lanex screen monitored by a CCD camera placed 426 mm behind the gas jet. In order to quantitatively measure the beam focusing, an Al plate with four 2 mm holes separated by 10 mm referred to as a “pepperpot” was inserted into the beam just after the magnetic lens restricting the electron propagation into four distinct beams. The result of the measured focusing power of the ring magnets was compared with the ray-tracing simulation with a good agreement. For focusing geometry, the spacing between the four electron beams was observed to decrease as the magnetic lens was moving downstream. This method was used to characterize the focusing power of the lenses and electron beam manipulation. The pepperpot was also used to verify the beam collimation with the two lens system. We have measured the magnetic field profile of the magnetic lenses and put this into a magnetic raytracing simulation, from which we have inferred its focal length, in the order of 40–80 cm for energies 1.5–2 MeV. With the same simulations, we have modelled the complex beampath consisting of the magnetic chicane and two ring magnets, and it predicted a close to parallel beam with similar profile as observed in the experiment.

### Charge determination

The electron charge measurement was carried out using the absolutely calibrated IP (Fujifilm MS) placed 267 cm behind the gas jet. Since the IP was located behind the magnetic lense setup, this charge was determined specifically for the selected narrow energy band used for the radiographic measurement. The image formed on the IP is scanned by the Fujifilm BAS 1800 scanner at 16 bit, with sensitivity setting of 4000, latitude of 5 and pixel size of 50 $\upmu$m. Each scan was performed 20 min after the laser shot. The charge is measured by integrating the signal on the Image Plate, converting tho PSL values (photo-stimulated luminescence) using the scanner characteristics, and then converting to deposited charge using the calibration in Tanaka et al.^32^. For a single electron energy, it is thus straightforward to integrate the full PSL count and convert that into the total electron charge measured by the IP. Using this calibration, we found the total electron charge to be 77 ± 47 pC in the collimated electron beam at the IP detector plane, i.e. after the magnetic lenses, 267 cm from the electron source. The electron spectrum for each shot was known. In the case of the radiographic measurement, a narrow band of electron energies was selected by the combination of the magnetic spectrometer coupled with the 5 mm slit and a set of two ring magnets that further selected and collimated the desired range of energies. Our estimate based on ray-tracing simulations and the edge enhancement of the structures in eRad images allowed us to estimate the electron energy bandwidth to be 1.9 ± 0.4 MeV. This is the final charge per shot usable for the eRad measurement. It should be noted that the total charge produced by the laser acceleration is higher as much of the beam was reduced by the slit and the magnetic lens beam path.

### PIC simulations

The electron acceleration was modelled by a 2D particle-in-cell (PIC) simulation code Epoch^27,28^. The simulation box had a size of 620 $\upmu$m $\times$ 40 $\upmu$m in the x–y plane. The corresponding mesh size is $\delta x = \delta y = 40$ nm. Thirtytwo quasiparticles per cell are employed with a total number of $4.96\times 10^8$. Both the electrons and ions had the initial temperature of 20 eV with a Maxwellian distribution. The plasma density had a Gaussian distribution in the longitudinal direction with the FWHM = 300 $\upmu$m and the peak density $10^{19}$ cm$^{-3}$. The laser parameters in the simulations were consistent with the experimental conditions. The laser beam interacted with a 600 $\upmu$m FWHM Gaussian gas density profile, which was a realistic model for most of the nozzles used. The free boundary conditions are applied in treating fields and particles.
